# Development of a robust predictive model for neutropenia after esophageal cancer chemotherapy using GLMMLasso

**DOI:** 10.1007/s11096-024-01836-5

**Published:** 2024-11-21

**Authors:** Shuhei Sugaya, Masashi Uchida, Takaaki Suzuki, Eiryo Kawakami, Itsuko Ishii

**Affiliations:** 1 Division of Pharmacy, Chiba University Hospital, Chiba, Japan; 2 Graduate School of Pharmaceutical Sciences, Chiba University, Chiba, Japan; 3 Advanced Data Science Project, RIKEN Information R&D and Strategy Headquarters, RIKEN, Yokohama, Kanagawa Japan; 4 Department of Artificial Intelligence Medicine, Graduate School of Medicine, Chiba University, Chiba, Japan; 5 Institute for Advanced Academic Research (IAAR), Chiba University, Chiba, Japan

**Keywords:** Esophageal cancer chemotherapy, GLMMLasso, Neutropenia, Nomogram

## Abstract

**Background:**

Neutropenia can easily progress to febrile neutropenia and is a risk factor for life-threatening infections. Predicting and preventing severe neutropenia can help avoid such infections.

**Aim:**

This study aimed to develop an optimal model using advanced statistical methods to predict neutropenia after 5-fluorouracil/cisplatin chemotherapy for esophageal cancer and to create a nomogram for clinical application.

**Method:**

Patients who received 5-fluorouracil/cisplatin chemotherapy at Chiba University Hospital, Japan, between January 2011 and March 2021 were included. Clinical parameters were measured before the first, second, and third chemotherapy cycles and were randomly divided by patient into a training cohort (60%) and test cohort (40%). The predictive performance of Logistic, Stepwise, Lasso, and GLMMLasso models was evaluated by the area under the receiver-operating characteristic curve (AUC). A nomogram based on GLMMLasso was developed, and the accuracy of probabilistic predictions was evaluated by the Brier score.

**Results:**

The AUC for the first cycle of chemotherapy was 0.781 for GLMMLasso, 0.751 for Lasso, 0.697 for Stepwise, and 0.669 for Logistic. The respective AUCs for GLMMLasso in the second and third cycles were 0.704 and 0.900. The variables selected by GLMMLasso were cisplatin dose, 5-fluorouracil dose, use of leucovorin, sex, cholinesterase, and platelets. A nomogram predicting neutropenia was created based on each regression coefficient. The Brier score for the nomogram was 0.139.

**Conclusion:**

We have developed a predictive model with high performance using GLMMLasso. Our nomogram can represent risk visually and may facilitate the assessment of the probability of chemotherapy-induced severe neutropenia in clinical practice.

**Supplementary Information:**

The online version contains supplementary material available at 10.1007/s11096-024-01836-5.

## Impact statements

- A generalized linear mixed model with least absolute shrinkage and selection operator (GLMMLasso) showed better prediction performance for severe neutropenia after 5-fluorouracil/cisplatin chemotherapy in the first and subsequent cycles than conventional statistical models.
- The variables selected by GLMMLasso were cisplatin dose, 5- fluorouracil dose, use of leucovorin, patient sex, cholinesterase, and platelets.
- The nomogram can easily calculate the risk of severe neutropenia induced by 5-fluorouracil/cisplatin chemotherapy in clinical practice.

## Introduction

Esophageal cancer is one of the most malignant cancers because of its aggressive nature and poor survival rate [1]. The combination of cisplatin (CDDP) and 5-fluorouracil (5-FU)—the FP chemotherapy regimen—is commonly used for neoadjuvant chemotherapy and for advanced esophageal cancer. This regimen is often combined with leucovorin to enhance the antitumor activity of 5-FU, which is also expected to increase toxicity [2]. One of the dose-limiting toxicities associated with FP chemotherapy is neutropenia, which increases a patient’s susceptibility to infections and can easily progress to febrile neutropenia. Predicting and preventing severe neutropenia can avoid life-threatening infections. Therefore, it is crucial to clarify the risk factors for neutropenia and construct a simple prediction tool.

Previous studies have found that older age, low platelet count, and hoarseness are risk factors for chemotherapy-induced neutropenia or febrile neutropenia in patients with esophageal cancer [3–5]. These findings are difficult to incorporate into routine clinical practice for several reasons. First, logistic regression was used to determine the risk factors in these studies. Logistic regression is widely used to estimate the effects of various predictors on binary outcomes but has potential issues with overfitting where it is more susceptible to minor changes or fluctuations in unseen data and tends to be poorly predictive when applied to other datasets [6]. For example, it has been reported that outliers in the data used can lead to erroneous estimation of coefficients and misclassification [7]. Second, the previous studies only analyzed the data for the first cycle of chemotherapy. In clinical practice, multiple cycles are often administered, and it could be preferable to use longitudinal data from multiple cycles to assess the effect of risk factors on the likelihood of neutropenia. Finally, although previous studies reported the odds ratios for individual risk factors, it is difficult to evaluate the specific probability of developing neutropenia in each patient using odds ratios.

Least absolute shrinkage and selection operator (Lasso) regression is an advanced variable selection algorithm for multivariate data. It is particularly well suited for dealing with large numbers of clinical factors and avoiding overfitting [8]. Furthermore, learning multiple cycles of longitudinal data through a generalized linear mixed model with Lasso penalization (GLMMLasso) makes it possible to evaluate the effects of changes in factors in each cycle. Moreover, nomograms are simple visual tools that can calculate the probability of an outcome. Although nomograms predicting neutropenia have been reported for irinotecan-based regimens in colorectal cancer and for docetaxel therapy in prostate cancer [9, 10], there have been no studies in esophageal cancer.

### Aim

This study had two aims. The first was to develop an optimal model to predict neutropenia after FP chemotherapy for esophageal cancer with high prediction performance that accounts for the effects of multiple variables. The second aim was to create a nomogram based on the optimal prediction model.

### Ethics approval

The study protocol was approved by the ethics committee of Chiba University Hospital on February 2, 2021 (approval number 4028).

## Method

### Patients and treatments

This cross-sectional survey included patients with esophageal cancer who received FP chemotherapy at Chiba University Hospital between January 2011 and March 2021. The purpose of this study was to create a predictive model and we did not specify study size. The medical data within 1 week before the first, second and third cycles of chemotherapy per patient were extracted from the electronic records system. The variables used in this study were routinely measured before chemotherapy. Patients with unmeasured variables were assumed to be missing completely at random and excluded. The FP regimen consisted of a 1-h intravenous infusion of CDDP at 80 mg/m^2^ and a continuous infusion of 5-FU at 800 mg/m^2^/day on days 1–5. If leucovorin was used, a 1-h intravenous infusion of 27 mg on days 1–5 was added to the FP regimen. Standard antiemetic support was provided, including an NK_1_ antagonist, a 5-HT_3_ antagonist, and a corticosteroid. A conventional or short hydration protocol for CDDP was used [11]. This regimen was repeated every 4 weeks. We extracted the lowest neutrophil count recorded within the 4 weeks after chemotherapy for each cycle, and neutropenia was defined as grade 4 neutropenia (i.e., a neutrophil count < 500 cells/μL) based on the National Cancer Institute Common Terminology Criteria for Adverse Events version 5.0.

### Variables selection and pre-processing

We retrospectively collected data on clinical parameters before chemotherapy, including age, sex, body surface area (BSA), CDDP dose (mg/m^2^), 5-FU dose (mg/m^2^), use of leucovorin, use of a short hydration protocol, aspartate aminotransferase, alanine aminotransferase, lactate dehydrogenase, gamma-glutamyl transpeptidase, cholinesterase, creatine kinase, amylase, total bilirubin, direct bilirubin, total protein, albumin, uric acid, blood urea nitrogen, creatinine, sodium, potassium, chloride, calcium, hemoglobin, hematocrit, platelets, C-reactive protein, neutrophils, eosinophils, basophils, monocytes, and lymphocytes. The dataset was randomly divided into a training cohort (60%) and a test cohort (40%).

### Statistical analysis

All analyses were performed using R software (version 4.2.0; The R Foundation for Statistical Computing, Vienna, Austria). Continuous variables were standardized to compare regression coefficients across models. Using the first cycle of chemotherapy in the training cohort, we developed three models, namely, logistic regression (Logistic), logistic regression after variable selection by stepwise method (Stepwise), and Lasso. For logistic regression, the “glm” function in the R stats package was used, and for Stepwise, variables were selected with the “step” function in the R stats package using the forward method based on the Akaike information criterion. In GLMMLasso implemented using the R glmmLasso package, we utilized a training cohort with repeated measurements from the first cycle to the third cycle [12]. Both Lasso and GLMMLasso required the optimal tuning parameter λopt. The “cv.glmnet” function in the R glmnet package and the Bayesian information criterion were used to determine the respective optimal λopt values for Lasso and GLMMLasso [13]. The prediction performance of each model was assessed using the area under the receiver-operating characteristic curve (AUC) for the test cohort.

We created a nomogram using selected variables and regression coefficients in the trained GLMMLasso. The rms package in R was used to draw nomograms and calibration plots. A nomogram was evaluated in terms of the accuracy of probabilistic predictions by the Brier score.

Patient characteristics were compared between the training and test cohorts using Fisher's exact test and the Mann–Whitney U test. A *p* value of < 0.01 was considered statistically significant.

## Results

### Patient characteristics

The patient characteristics are shown in Table 1. A total of 366 patients who received 624 cycles of FP chemotherapy were included and divided into a training cohort (n = 219 [385 cycles]) and a test cohort (n = 147 [239 cycles]) (Table 1).

**Table 1:** Patient characteristics

	Cycle 1			Cycle 2			Cycle 3		
	Training cohort (n = 219)	Test cohort (n = 147)	*p* value	Training cohort (n = 115)	Test cohort (n = 65)	*p* value	Training cohort (n = 51)	Test cohort (n = 27)	*p* value
Age (years)	69 (62, 74)	70 (65, 76)	0.21^b^	69 (62, 74)	73 (66, 77)	0.05^b^	71 (63, 74)	72 (68, 76)	0.26^b^
Sex (female) (%)	29 (13)	24 (16)	0.45^a^	13 (11)	10 (15)	0.49^a^	7 (14)	4 (15)	1^a^
Body surface area (m^2^)	1.6 (1.5, 1.7)	1.6 (1.5, 1.7)	0.61^b^	1.6 (1.5, 1.7)	1.6 (1.5, 1.7)	0.79^b^	1.6 (1.5, 1.7)	1.6 (1.5, 1.7)	0.77^b^
CDDP (mg/m^2^)	79 (74, 80)	78 (64, 80)	0.14^b^	66 (63, 80)	64 (63, 78)	0.08^b^	64 (63, 80)	64 (57, 65)	0.11^b^
5-fluorouracil (mg/m^2^)	779 (762, 790)	779 (755, 792)	0.81^b^	757 (619, 788)	657 (621, 788)	0.65^b^	634 (593, 780)	629 (619, 732)	0.99^b^
Leucovorin use (yes) (%)	114 (52)	82 (56)	0.52^a^	42 (37)	19 (29)	0.41^a^	18 (35)	5 (19)	0.19^a^
Short hydration (yes) (%)	121 (55)	76 (52)	0.52^a^	87 (76)	50 (77)	1^a^	45 (88)	24 (89)	1^a^
Aspartate aminotransferase (IU/L)	21 (17, 28)	21 (17, 25)	0.59^b^	19 (15, 25)	19 (16, 24)	0.93^b^	19 (16, 25)	20 (17, 30)	0.4^b^
Alanine aminotransferase (IU/L)	17 (12, 29)	16 (13, 23)	0.27^b^	14 (10, 23)	14 (11, 18)	0.81^b^	15 (11, 19)	13 (11, 17)	0.66^b^
Lactate dehydrogenase (IU/L)	180 (157, 217)	186 (165, 214)	0.25^b^	187 (166, 214)	189 (161, 214)	0.8^b^	188 (162, 208)	199 (171, 231)	0.22^b^
Gamma-glutamyl transpeptidase (IU/L)	37 (23, 68)	35 (25, 59)	0.78^b^	37 (23, 65)	36 (29, 73)	0.64^b^	33 (24, 67)	46 (28, 90)	0.11^b^
Cholinesterase (IU/L)	235 (200, 273)	243 (203, 295)	0.12^b^	226 (201, 264)	252 (215, 278)	0.05^b^	239 (211, 293)	258 (227, 294)	0.45^b^
Creatine kinase (IU/L)	52 (33, 82)	56 (39, 81)	0.18^b^	49 (34, 67)	64 (43, 89)	0.01^b^	51 (40, 75)	88 (55, 128)	0.01^b^
Amylase (IU/L)	73 (56, 97)	71 (52, 96)	0.32^b^	81 (56, 101)	81 (57, 93)	0.8^b^	83 (62, 106)	77 (54, 95)	0.42^b^
Total bilirubin (mg/dL)	0.6 (0.4, 0.8)	0.6 (0.5, 0.8)	0.82^b^	0.5 (0.4, 0.6)	0.6 (0.4, 0.7)	0.08^b^	0.5 (0.4, 0.7)	0.6 (0.45, 0.8)	0.13^b^
Direct bilirubin (mg/dL)	0.1 (0, 0.1)	0.1 (0.1, 0.1)	0.71^b^	0.1 (0, 0.1)	0.1 (0, 0.1)	0.02^b^	0.1 (0, 0.1)	0.1 (0.1, 0.1)	0.04^b^
Total protein (g/dL)	6.6 (6.3, 7)	6.6 (6.4, 7)	0.44^b^	6.5 (6, 6.8)	6.6 (6.2, 6.8)	0.16^b^	6.6 (6.2, 6.8)	6.6 (6.3, 6.9)	0.62^b^
Albumin (mg/dL)	3.7 (3.3, 4)	3.7 (3.4, 4.1)	0.38^b^	3.6 (3.3, 3.9)	3.7 (3.4, 4)	0.21^b^	3.8 (3.5, 3.9)	3.8 (3.6, 4.1)	0.67^b^
Uric acid (mg/dL)	4.9 (3.7, 5.9)	4.9 (4.1, 6)	0.26^b^	4.7 (3.6, 5.6)	5 (4.4, 5.8)	0.03^b^	5 (4.2, 6)	5.3 (4.9, 6.1)	0.17^b^
Blood urea nitrogen (mg/dL)	14 (11, 17)	14 (11, 17)	0.9^b^	15 (12, 17)	15 (13, 19)	0.34^b^	15 (13, 18)	15 (13, 19)	0.97^b^
Creatinine (mg/dL)	0.73 (0.63, 0.84)	0.75 (0.63, 0.89)	0.14^b^	0.76 (0.66, 0.91)	0.78 (0.68, 0.88)	0.78^b^	0.74 (0.67, 0.89)	0.86 (0.73, 0.98)	0.11^b^
Sodium (mEq/L)	139 (137, 140)	139 (138, 141)	0.12^b^	140 (138, 141)	140 (138, 142)	0.47^b^	140 (138, 141)	141 (141, 142)	0.01^b^
Potassium (mEq/L)	4.3 (4.1, 4.6)	4.2 (4.1, 4.5)	0.09^b^	4.3 (4.1, 4.5)	4.3 (4.1, 4.5)	0.75^b^	4.4 (4.2, 4.7)	4.2 (4.1, 4.4)	0.07^b^
Chloride (mEq/L)	104 (102, 106)	105 (103, 107)	0.06^b^	105 (103, 107)	106 (103, 107)	0.41^b^	106 (104, 107)	106 (105, 108)	0.25^b^
Calcium (mg/dL)	9.1 (8.8, 9.4)	9.1 (8.8, 9.4)	0.71^b^	9 (8.7, 9.3)	9 (8.7, 9.4)	0.83^b^	9.1 (8.9, 9.5)	9 (8.9, 9.3)	0.32^b^
Hemoglobin (g/dL)	12 (11, 13)	12 (12, 13)	0.16^b^	11 (10, 12)	12 (11, 12)	0.19^b^	11 (10, 12)	12 (11, 12)	0.55^b^
Hematocrit (%)	37 (33, 40)	37 (35, 40)	0.2^b^	34 (31, 37)	35 (31, 37)	0.31^b^	34 (31, 37)	35 (32, 37)	0.74^b^
Platelets (× 1000/μL)	234 (186, 292)	218 (177, 278)	0.04^b^	240 (189, 298)	223 (182, 270)	0.14^b^	222 (191, 272)	225 (159, 253)	0.24^b^
C-reactive protein (mg/dL)	0.3 (0.1, 1.4)	0.3 (0.1, 1.1)	0.99^b^	0.21 (0.085, 1)	0.1 (0.05, 0.6)	0.18^b^	0.16 (0.05, 0.5)	0.2 (0.07, 0.71)	0.45^b^
Neutrophils (/μL)	3790 (2730, 5340)	3920 (3130, 5410)	0.33^b^	3460 (2620, 5470)	3550 (2760, 4480)	0.43^b^	3850 (2740, 4930)	2950 (2160, 4220)	0.04^b^
Eosinophils (/μL)	111 (61, 199)	119 (69, 182)	0.85^b^	56 (21, 133)	55 (27, 111)	0.77^b^	62 (37, 129)	72 (20, 147)	0.97^b^
Basophils (/μL)	21 (11, 39)	21 (12, 31)	0.84^b^	22 (12, 39)	22 (12, 39)	0.78^b^	23 (13, 40)	21 (11, 41)	0.9^b^
Monocytes (/μL)	420 (301, 549)	403 (310, 528)	0.97^b^	428 (334, 551)	405 (330, 510)	0.37^b^	429 (338, 586)	360 (274, 438)	0.05^b^
Lymphocytes (/μL)	1050 (761, 1470)	1150 (801, 1500)	0.25^b^	1030 (705, 1490)	1140 (726, 1530)	0.24^b^	956 (710, 1460)	979 (674, 1330)	0.87^b^
Grade 4 neutropenia (yes) (%)	44 (20)	45 (31)	0.53^a^	10 (9)	3 (5)	0.38^a^	4 (8)	2 (7)	1^a^

Descriptive statistics are shown as the median (interquartile range)

^a^Fisher’s exact test; ^b^Mann–Whitney* U* test

### Developing the predictive models in the training cohort

**Fig. 1:**
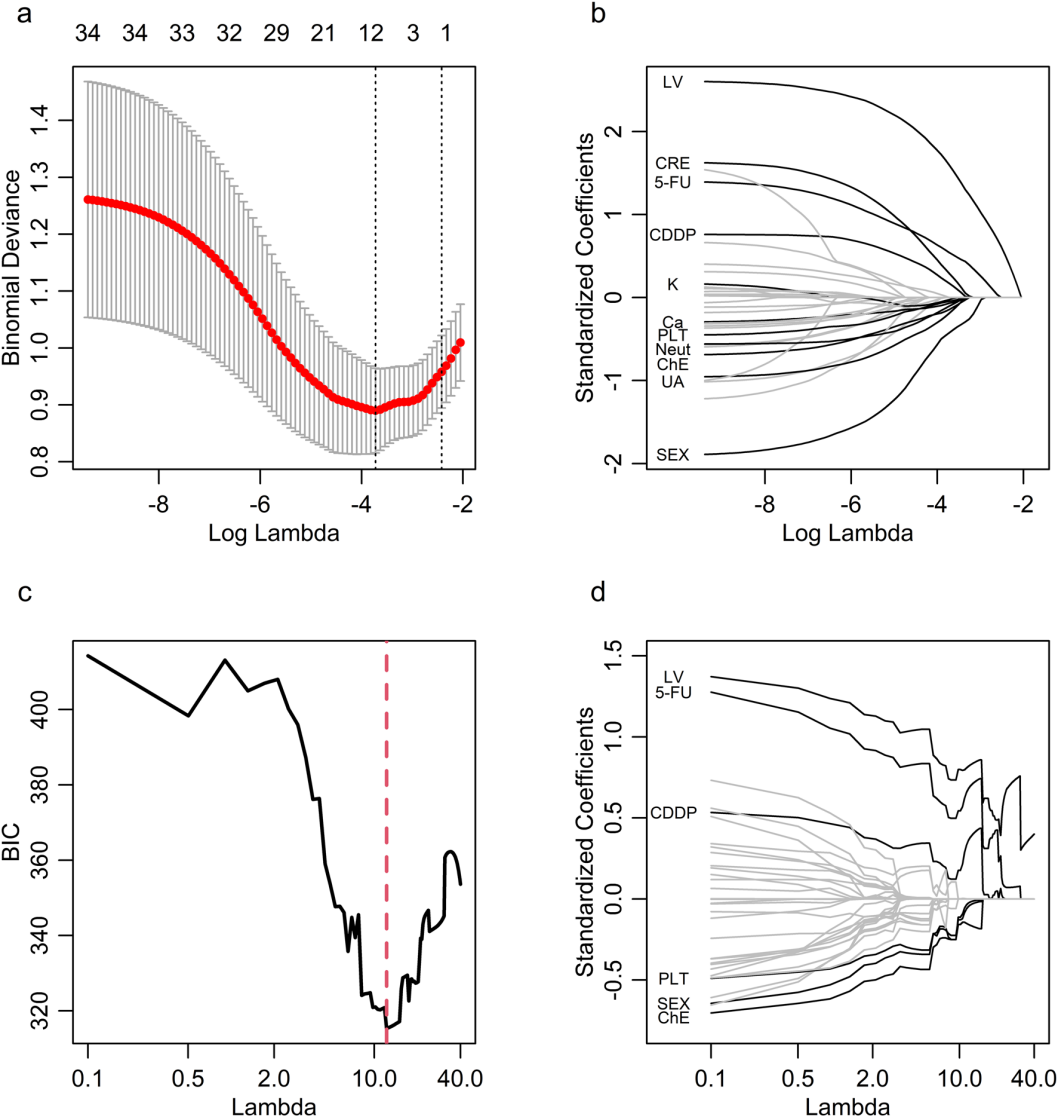
Regularization path for Lasso and GLMMLasso. **a** Deviance of the Lasso for determination of the optimal tuning parameter, λopt. Two specific values are indicated by the vertical dotted lines. Lambda.min gives the minimum mean cross-validated error, while lambda.1se gives the most regularized model such that the cross-validated error is within one standard error of the minimum. We used lambda.min as λopt. **b** Coefficient paths for the Lasso. **c** Bayesian information criterion of the GLMMLasso for determination of the optimal tuning parameter λopt. **d** Coefficient paths for the GLMMLasso. BIC, Bayesian information criterion; Lasso, least absolute shrinkage and selection operator

We developed four models, namely, Logistic, Stepwise, Lasso, and GLMMLasso. Eleven variables were selected by Stepwise: sex, BSA, leucovorin, CDDP dose, 5-FU dose, lactate dehydrogenase, cholinesterase, uric acid, creatinine, calcium, and neutrophils. The Lasso analysis determined the optimal tuning parameter (λ) value of 0.024 with log (λ) =  − 3.72 (Fig. 1a), and eleven variables were selected, including sex, leucovorin, CDDP dose, 5-FU dose, cholinesterase, uric acid, creatinine, potassium, calcium, platelets, and neutrophils (Fig. 1b). For the GLMMLasso method, an optimal value of λ = 11.38 was obtained (Fig. 1c), and six variables were selected: sex, leucovorin, CDDP dose, 5-FU dose, cholinesterase, and platelets (Fig. 1d).

**Fig. 2:**
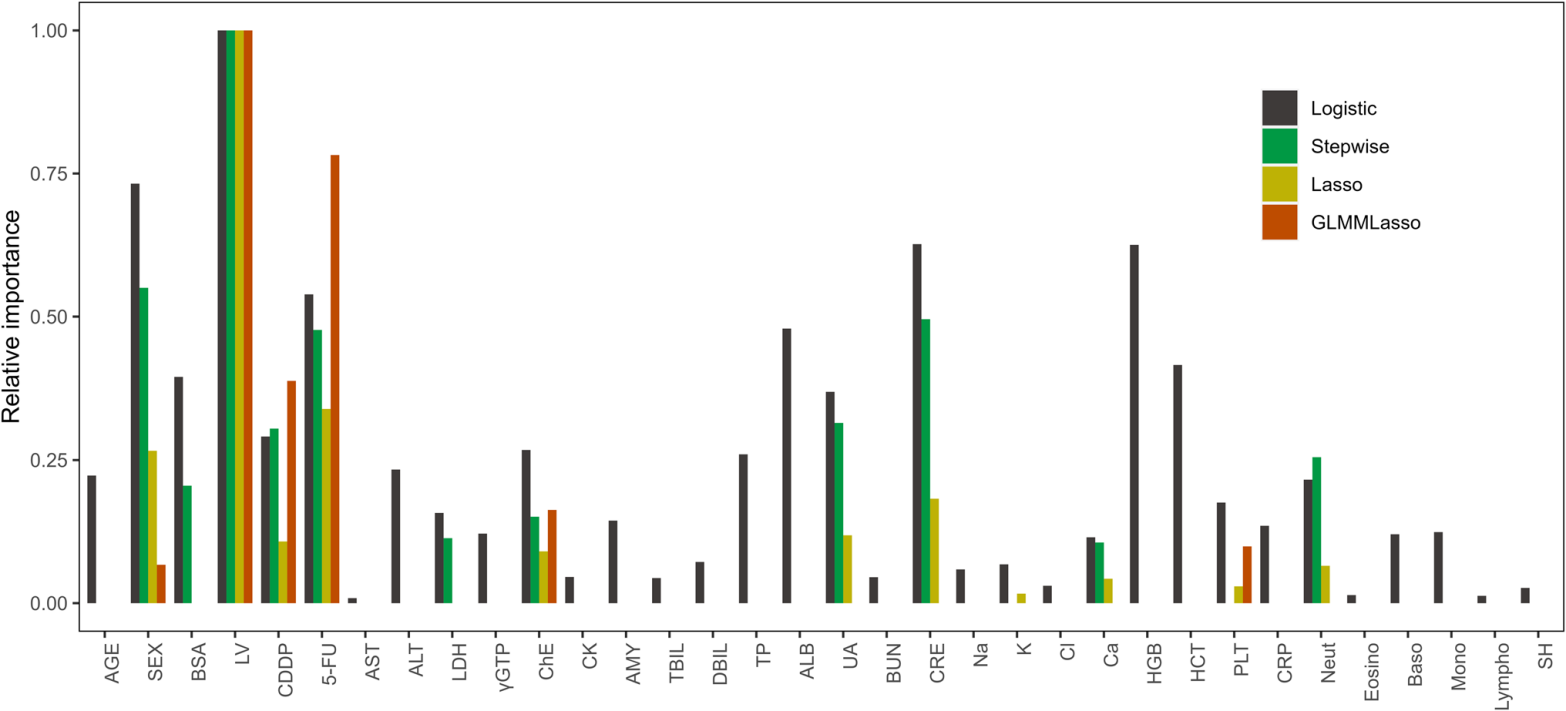
Relative importance of variables for prediction of neutropenia calculated in the Logistic, Stepwise, Lasso, and GLMMLasso models. The importance of the variables is represented as a ratio of the highest value (i.e., leucovorin). γGTP, gamma-glutamyl transpeptidase; 5-FU, 5-fluorouracil; ALB, albumin; ALT, alanine aminotransferase; AMY, amylase; AST, aspartate aminotransferase; Baso, basophils; BSA body surface area; BUN, blood urea nitrogen; Ca, calcium; CDDP, cisplatin; ChE, cholinesterase; CK, creatine kinase; Cl, chloride; CRE, creatinine; CRP, C-reactive protein; DBIL, direct bilirubin; Eosino, eosinophils; HCT, hematocrit; HGB, hemoglobin; K, potassium; LDH, lactate dehydrogenase; LV, leucovorin; Lympho, lymphocytes; Mono, monocytes; Na, sodium; Neut, neutrophils; PLT, platelets; SH, short hydration protocol; TBIL, total bilirubin; TP, total protein; UA, uric acid

Each regression coefficient is shown as a standardized coefficient (Table 2). Relative importance was defined as the ratio of the absolute value of the coefficient with the highest value (Fig. 2). Nine of the variables selected by the Stepwise and Lasso models were the same and tended to have lower relative importance in Lasso. Among the variables selected by GLMMLasso, the doses of CDDP and 5-FU had higher relative importance than in Stepwise and Lasso. The variables selected by all models were sex, leucovorin, CDDP dose, 5-FU dose, and cholinesterase. Men tended to receive higher doses of 5-FU than women in the group that also received leucovorin, although the difference was not statistically significant (*p* = 0.14) (Supplementary Fig. 1).

**Table 2:** Standardized coefficients for the four models

	Logistic	Stepwise	Lasso	GLMMLasso
Age	− 0.58			
Sex (female)	− 1.9	− 1.5	− 0.43	− 0.053
Body surface area	− 1	− 0.55		
Leucovorin use (yes)	2.6	2.7	1.6	0.79
CDDP	0.76	0.81	0.17	0.3
5-flurouracil	1.4	1.3	0.54	0.61
Aspartate aminotransferase	0.023			
Alanine aminotransferase	− 0.61			
Lactate dehydrogenase	0.41	0.3		
Gamma-glutamyl transpeptidase	0.32			
Cholinesterase	− 0.7	− 0.4	− 0.14	− 0.13
Creatine kinase	− 0.12			
Amylase	− 0.38			
Total bilirubin	0.11			
Direct bilirubin	− 0.19			
Total protein	0.68			
Albumin	− 1.3			
Uric acid	− 0.96	− 0.84	− 0.19	
Blood urea nitrogen	0.12			
Creatinine	1.6	1.3	0.29	
Sodium	0.15			
Potassium	0.18		− 0.026	
Chloride	− 0.079			
Calcium	− 0.3	− 0.28	− 0.068	
Hemoglobin	1.6			
Hematocrit	− 1.1			
Platelets	− 0.46		− 0.047	− 0.078
C-reactive protein	− 0.35			
Neutrophils	− 0.56	− 0.68	− 0.1	
Eosinophils	0.037			
Basophils	− 0.31			
Monocytes	− 0.32			
Lymphocytes	0.033			
Short hydration (yes)	0.07

### Prediction performance

**Fig. 3:**
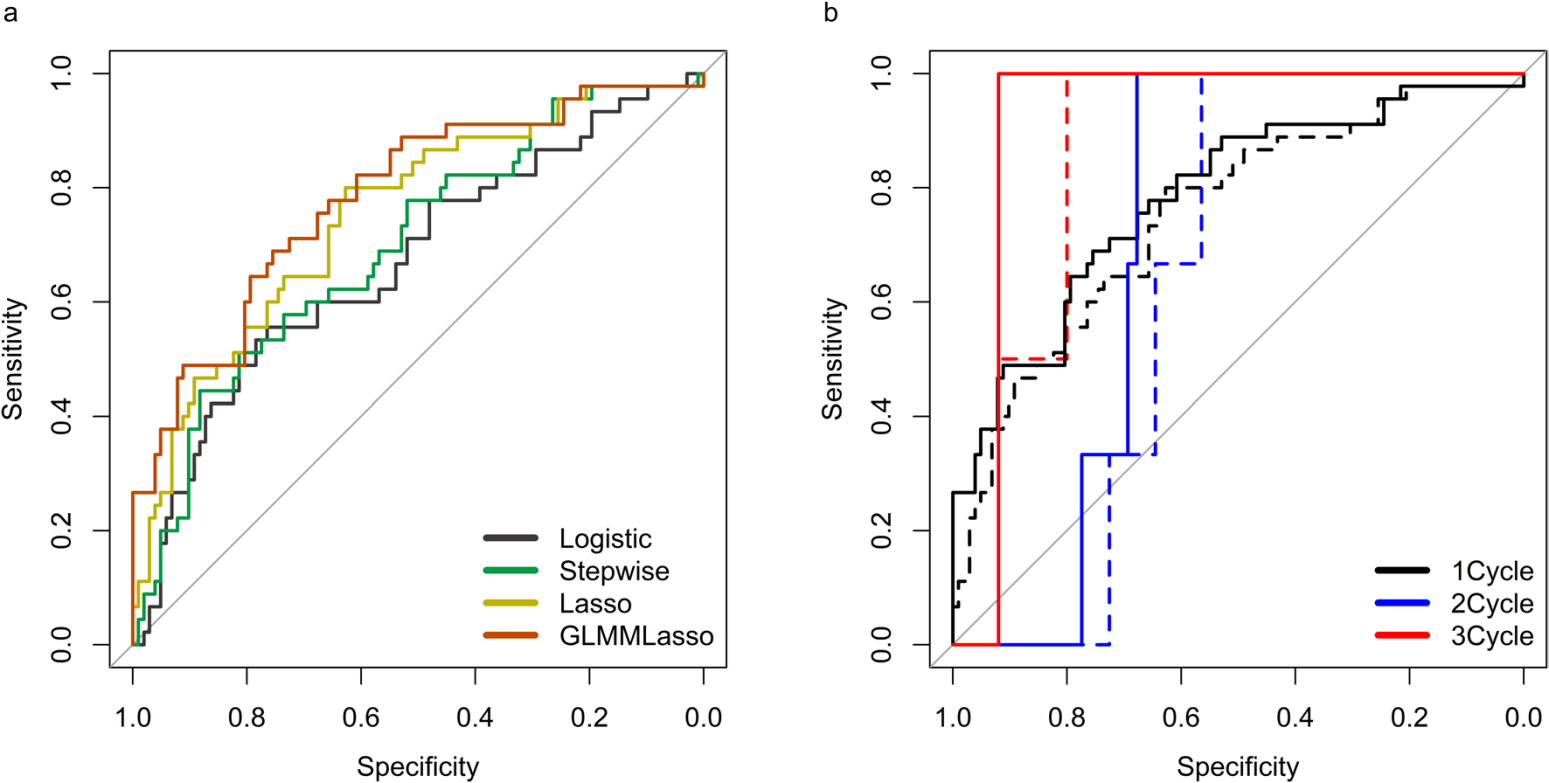
Validation of the prediction performance of each model. **a** Receiver-operating characteristic curves derived from the Logistic, Stepwise, Lasso and GLMMLasso models. **b** Receiver-operating characteristic curves derived from Lasso and GLMMLasso for the first, second, and third cycles of chemotherapy. Dashed lines represent Lasso. Solid lines represent GLMMLasso

We assessed the AUC for the first cycle in the test cohort using the Logistic, Stepwise, Lasso, and GLMMLasso models (Fig. 3a). The AUC was 0.781 for GLMMLasso, 0.751 for Lasso, 0.697 for Stepwise, and 0.669 for Logistic, with GLMMLasso showing the best performance. In the test cohort, the AUCs for the first, second, and third cycles of chemotherapy were 0.781, 0.704, and 0.900, respectively, for GLMMLasso and 0.751, 0.645, and 0.860 for Lasso (Fig. 3b). The GLMMLasso model had better predictive performance than the Lasso model in all cycles.

### Construction of the nomogram

**Fig. 4:**
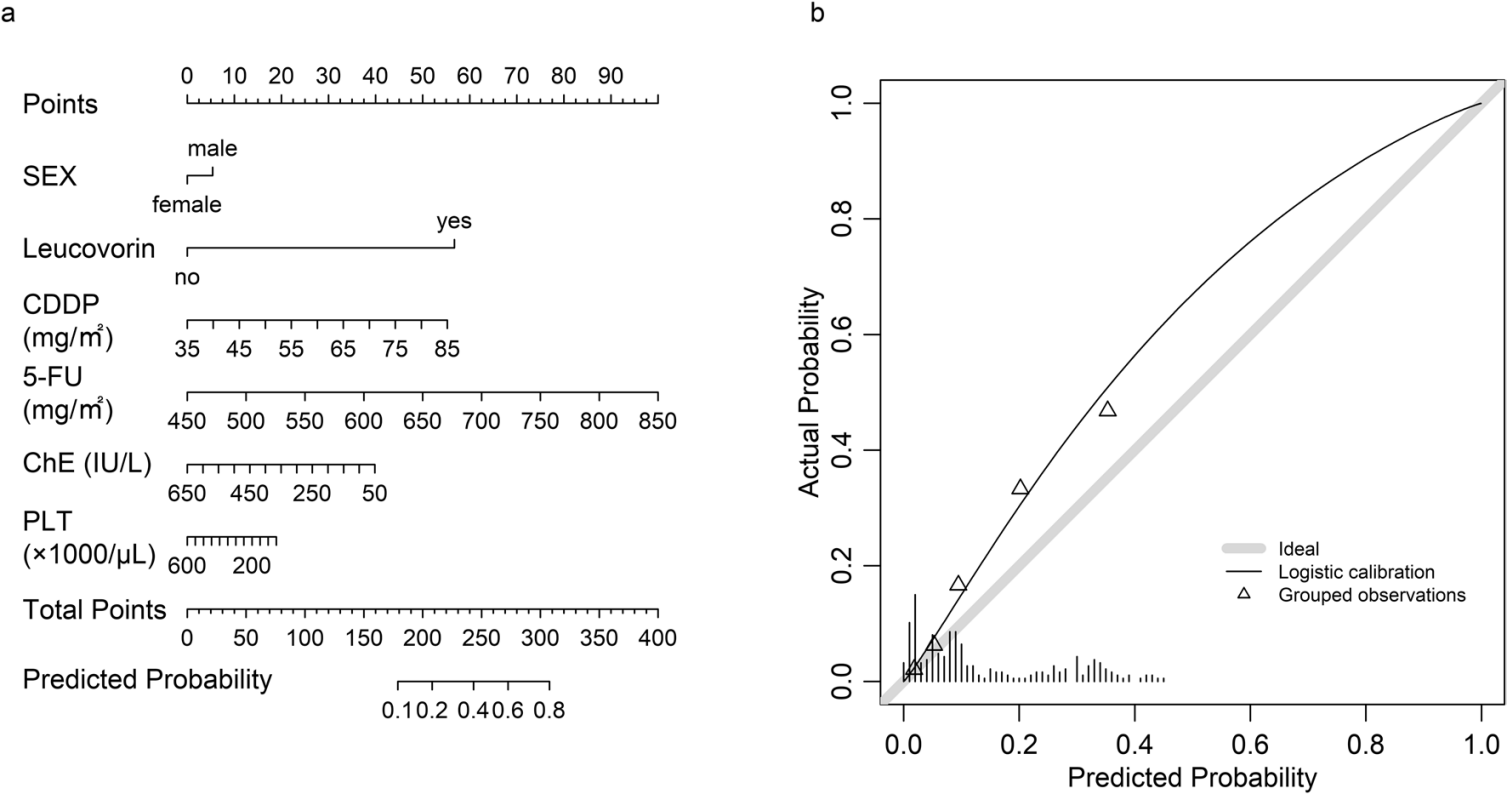
Nomogram and the calibration plot for the GLMMLasso model. **a** Nomogram predicting the risk of neutropenia in the training cohort. **b** Calibration plot for the nomogram of the test cohort. The x-axis is the predicted probability of neutropenia and the y-axis is the actual probability. 5-FU, 5-fluorouracil; CDDP, cisplatin; ChE, cholinesterase; PLT, platelets

Figure 4a shows the nomogram constructed using the coefficients for each variable obtained by GLMMLasso. The nomogram is used as follows. First, a perpendicular line is drawn from the value of each variable to the “Points” axis and the corresponding value is found. Second, the sum of these values is marked on the “Total points” axis. Third, a perpendicular line is drawn to the “Predicted Probability” axis and the corresponding value is found. This value is the estimated probability of neutropenia.

Calibration plots in the test cohort were also generated for validation of the nomogram (Fig. 4b). The nomogram had a Brier score of 0.139.

## Discussion

In this study, we tested the ability of four models (Logistic, Stepwise, Lasso, and GLMMLasso) to predict neutropenia after FP chemotherapy. The AUC was slightly better with the Stepwise model, which used selected variables, than with the Logistic model, which used all variables. Furthermore, even though selection of variables with the Lasso model was similar to that with the Stepwise model, the AUC was higher with Lasso. Regularization prevents the coefficient estimates from becoming inappropriately large. This leads to the estimates being more stable and less sensitive to small changes in the data and therefore being more generalizable to external data [6, 8]. In this study, as regularization weakened, the regression coefficients increased and the deviance of the model decreased, which suggests overfitting (Fig. 1a). The relative importance of Lasso was lower than Stepwise for several variables (Fig. 2). This suggests that the predictive performance was higher for Lasso than for Stepwise because generalizability was improved by regularization. GLMMLasso showed better prediction accuracy than Lasso for the first, second, and third cycles in the test cohort. Moreover, the relative importance of 5-FU and CDDP doses was higher with GLMMLasso than with Lasso (Fig. 2). Neutropenia was less common in the second and third cycles probably because the doses of anticancer agents administered in those cycles were lower than those used in the first cycle. The relative importance of 5-FU and CDDP doses could be more accurately evaluated by including the second and third cycles as well as the first cycle in the study data.

Previous studies have found that older age, low platelet count, and hoarseness are risk factors for chemotherapy-induced neutropenia or febrile neutropenia in patients with esophageal cancer [3–5]. In addition, gender, leucovorin use, and various serum biomarkers such as albumin, hemoglobin, monocytes, and lactate dehydrogenase have been reported as predictors of chemotherapy-induced neutropenia or febrile neutropenia in other cancer types [14–17]. Moreover, a previous study reported that it was possible to develop models with high diagnostic and prognostic accuracy by using a combination of biomarkers that can be obtained in routine practice [18]. Therefore, we developed predictive models using known possible risk factors and various biomarkers obtained in clinical practice. The variables selected by GLMMLasso were CDDP dose, 5-FU dose, use of leucovorin, patient sex, cholinesterase, and platelets. Neutropenia is a dose-limiting toxicity of CDDP and 5-FU, and our study found that the incidence of neutropenia tended to increase in a dose-dependent manner with these two agents. Leucovorin enhances inhibition of thymidylate synthase by biochemical modulation of 5-FU [19]. Administration of leucovorin has previously been reported to be a risk factor for grade 3 or higher leukopenia, diarrhea, and oral mucositis in patients treated with 5-FU [17], which is consistent with our present findings. Another study found that clearance of 5-FU is lower in women than in men and that women are more likely to develop severe fluoropyrimidine-related toxicity [20]. However, the opposite trend was observed in our present study, possibly because male patients who received leucovorin tended to receive higher doses of 5-FU than female patients (Supplementary Fig. 1). In our study, the small number of female patients may have introduced bias, so our findings require confirmation in a larger sample size.

Several studies have identified a low platelet count as a risk factor for neutropenia or febrile neutropenia [3, 21, 22]. Multivariate analysis in another study demonstrated a correlation between the pre-transplant platelet count and the yield of CD34 + cells in patients undergoing autologous peripheral blood stem cell transplantation [23]. Univariate analysis in a further study showed that the pre-transplant platelet count before high-dose chemotherapy was significantly associated with subsequent recovery of white blood cells [24]. Therefore, a low platelet count may predict patients who are more likely to experience delayed neutrophil recovery and develop neutropenia after chemotherapy.

Cholinesterase is a protein synthesized in the liver and known to diminish in malnourished patients because of reduced provision of nutrients to the liver [25]. In a rat model, a group on a low-protein diet showed diminished dihydropyrimidine dehydrogenase activity and reduced clearance of 5-FU when compared with a group on a normal diet [26]. This finding suggests that malnutrition increases the risk of 5-FU-induced neutropenia. Albumin is known to be a biomarker of nutritional status, but a recent report indicated that albumin serves primarily as an indicator of inflammation rather than nutritional status [27]. Cholinesterase may be less influenced by inflammation than albumin and is considered to be a more reliable indicator of malnutrition [25]. An investigation of nutritional status at 3, 6, and 12 months after surgery for esophageal cancer found no alterations in albumin levels but decreases in body mass index and cholinesterase at 3 months after surgery, followed by gradual recovery [28]. Cholinesterase may reflect malnutrition resulting from decreased dietary intake more precisely than albumin. As esophageal cancer progresses, oral consumption becomes increasingly difficult, often leading to malnutrition and weight loss. Although the present study could not evaluate dietary intake, a low cholinesterase level may correlate with reduced food intake and be a risk factor for neutropenia.

We have developed a nomogram that can predict neutropenia in clinical practice. The model calibration plots showed good prediction performance with a Brier score of 0.139. Our nomogram allows the probability of neutropenia to be predicted visually. In daily practice, physician or pharmacist calculates the predicted probability using the predictors (i.e., sex, leucovorin use, doses of anticancer agents, cholinesterase, and platelets) before the first, second, and third cycles. If the patient has a probability above a certain level, this may lead to proactive interventions, including dose reduction of anticancer agents and prophylactic administration of oral antimicrobial agents. These interventions have the potential to contribute to prevention of severe neutropenia. On the other hand, because the relationship between dose reduction and efficacy including prognosis is not clear, careful consideration should be given to dose modification.

This study has several limitations. First, it had a retrospective design, which limited the information that could be collected for accurate prediction of neutropenia. For example, hoarseness has been reported to be a risk factor for neutropenia but could not be investigated owing to insufficient documentation in the electronic medical records [3]. Predictive performance could be improved by incorporating information on genetic polymorphisms, such as those involving dihydropyrimidine dehydrogenase, and/or the blood concentration of anticancer drug. Second, all the study data came from a single center and may be overfitted to our dataset. Therefore, evaluation of the predictive performance of our nomogram using a large external dataset would be desirable. Third, we did not consider the effect of the cumulative CDDP dose from the first to third cycles in the GLMMLasso analysis (with a maximum cumulative CDDP dose of 240 mg/m^2^). Given the reports indicating no statistically significant difference in the incidence of neutropenia according to whether the CDDP doses received are more or less than 200 mg/m^2^ [29, 30], we assumed that the cumulative CDDP dose did not affect the incidence of neutropenia between the first and third cycles in this study. A sufficient number of samples per cycle should be collected and analyzed to account for the effect of cumulative dosing.

## Conclusion

We have developed a predictive model with high performance that can be applied to the first and subsequent cycles in contrast to conventional logistic regression. Our nomogram calculates the risk visually and may facilitate assessment of the probability of severe neutropenia induced by chemotherapy for esophageal cancer in clinical practice.

## Supplementary Information

Below is the link to the electronic supplementary material.

Supplementary file1 (DOCX 132 KB)
