# Supplementary material for: Development of a robust predictive model for neutropenia after esophageal cancer chemotherapy using GLMMLasso
Source: Int J Clin Pharm. 2024 Nov 21;47(4):990–1000. doi: 10.1007/s11096-024-01836-5 (PMC12335393; doi:10.1007/s11096-024-01836-5)

***Development of a robust predictive model for neutropenia after esophageal cancer chemotherapy***

***using GLMMLasso***

Shuhei Sugaya^1,2^, Masashi Uchida^1†^, Takaaki Suzuki^1,2^, Eiryo Kawakami^3,4,5^, Itsuko Ishii^1,2^

^1^ Division of Pharmacy, Chiba University Hospital, Chiba, Japan

^2^ Graduate School of Pharmaceutical Sciences, Chiba University, Chiba, Japan

^3^ Advanced Data Science Project, RIKEN Information R&D and Strategy Headquarters, RIKEN, Kanagawa, Japan

^4^ Department of Artificial Intelligence Medicine, Graduate School of Medicine, Chiba University, Chiba, Japan

^5^ Institute for Advanced Academic Research (IAAR), Chiba University, Chiba, Japan

**Supplementary Fig. 1** Doses of 5-fluorouracil in men and women receiving leucovorin. p = 0.14, Mann–Whitney *U* test.


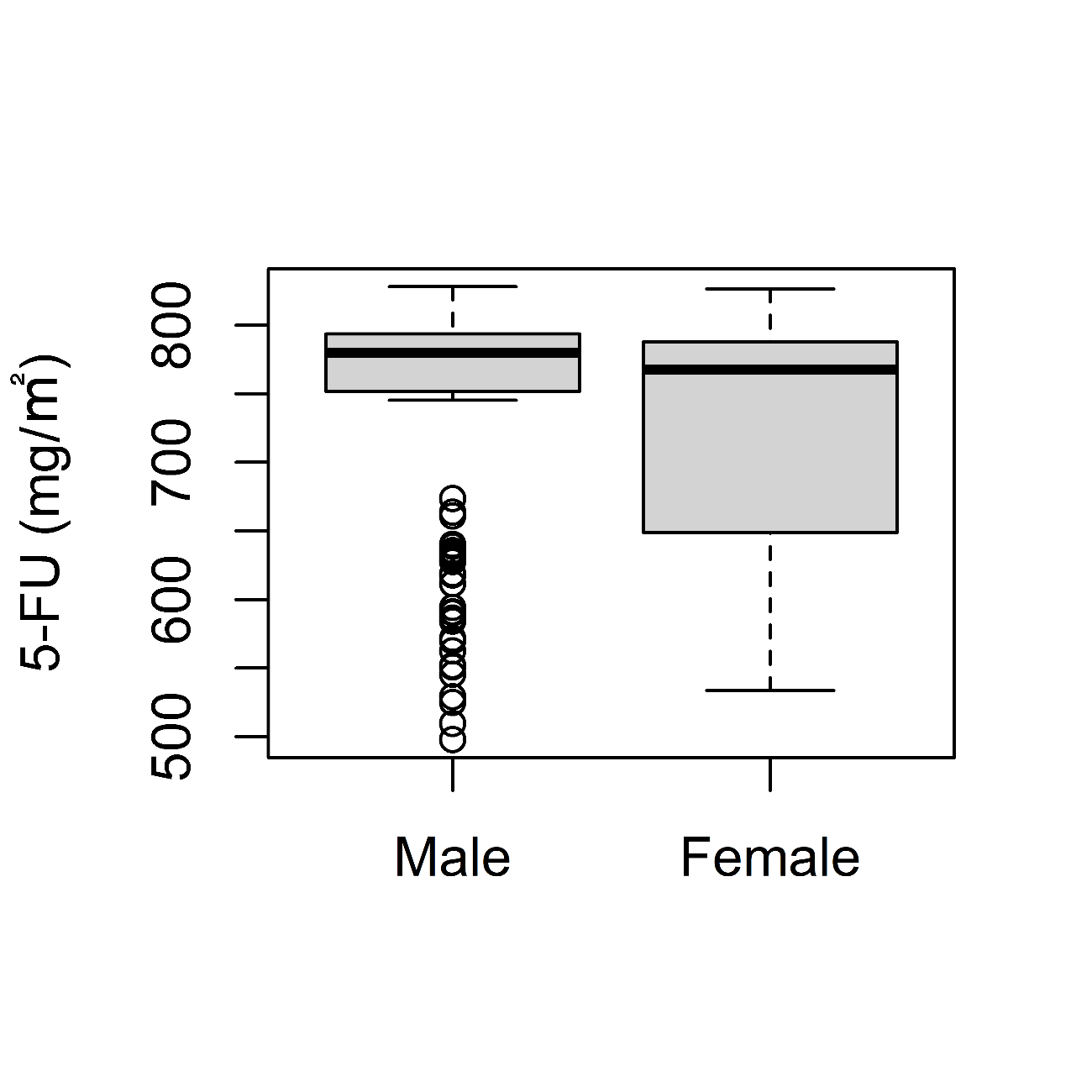

Supplement: Supplementary file 1 — Supplementary file1 (DOCX 132 KB) [file 11096_2024_1836_MOESM1_ESM.docx]
